# Factors involved in the delay of treatment initiation for cervical cancer patients: A nationwide population-based study

**DOI:** 10.1097/MD.0000000000004568

**Published:** 2016-08-19

**Authors:** Szu-Ching Shen, Yao-Ching Hung, Pei-Tseng Kung, Wen-Hui Yang, Yueh-Hsin Wang, Wen-Chen Tsai

**Affiliations:** a Department of Public Health; b Department of Health Services Administration, China Medical University, Taichung; c Department of Medical Affairs, Buddhist Dalin Tzu Chi Hospital, Chiayi; d Department of Health Services Administration, Chia Nan University of Pharmacy and Science, Tainan; e Department of Obstetrics and Gynecology, China Medical University Hospital; f Graduate Institution of Clinical Medical Science, School of Medicine, China Medical University; g Department of Healthcare Administration, Asia University, Taichung, Taiwan.

**Keywords:** cancer treatment, cervical cancer, delayed treatment, survival, universal health insurance

## Abstract

Cervical cancer ranks as the fourth leading cause of cancer death in women worldwide. In Taiwan, although the universal health insurance system has achieved 99.9% coverage and ensured easy access to medical care, some cervical cancer patients continue to delay initiation of definitive treatment after diagnosis. This study focused on cervical cancer patients who delayed treatment for at least 4 months, and examined the characteristics, related factors, and survival in these patients.

Data on patients with a new confirmed diagnosis of cervical cancer by the International Federation of Gynecology and Obstetrics (FIGO) staging system between 2005 and 2010 were obtained from the National Health Insurance Research Database and the Taiwan Cancer Registry. Logistic regression analysis was performed to analyze the association of various factors with treatment delay. The Cox proportional hazards model was used to analyze the effects of various factors on mortality risk.

The rate of treatment delay for cervical cancer decreased steadily from 6.46% in 2005 to 2.48% in 2010. Higher rates of treatment delay were observed among patients who were aged ≥75 years (9.91%), had severe comorbidity, had stage IV (9.50%), diagnosing hospital level at nonmedical center, or at public hospital ownership. Factors that correlated with treatment delay were age ≥75 years (odds ratio [OR] = 2.42), higher comorbidity Charlson comorbidity index (CCI) 4–6, or ≥7 (OR = 1.60, 2.00), cancer stage IV (OR = 2.60), the diagnosing hospital being a regional, district hospital, or other (OR = 3.00, 4.01, 4.60), and at public hospital ownership. Those who delayed treatment had 2.31 times the mortality risk of those who underwent timely treatment (*P* < 0.05).

Delayed cervical cancer treatment in Taiwan was associated with age, comorbidity, cancer stage, diagnosing hospital level, and hospital ownership. Delaying treatment for ≥4 months substantially raised mortality risk in cervical cancer patients.

## 1 Introduction

Cervical cancer is one of the five most common causes of cancer death in women worldwide, responsible for over 265,000 deaths in 2012, accounting for approximately 7% of cancer deaths in women.^[1]^ It ranks as the fourth leading cause of death from cancer in women.^[2]^ In Taiwan, approximately 1567 women were diagnosed with cervical cancer and over 640 died of the disease in 2014, making cervical cancer the seventh most common cause of cancer death among Taiwanese women and a significant health concern in Taiwan.^[3]^ In 1995, Taiwan's Ministry of Health and Welfare began providing one Pap smear test per year to every woman aged 30 years or older, and this measure has demonstrated remarkable efficacy against cervical cancer. Among the age-standardized incidence rates for the 10 leading cancers in women from 2002 to 2011, cervical cancer (41.4%) had the highest decrease rate.^[3]^ The age-standardized mortality from cervical cancer fell from 11 to 4 per 100,000 population between 1995 and 2013, a 64% reduction.^[3]^

Among pregnant patients with cervical cancer, consideration for better fetal outcome may be a reason for choosing to delay treatment.^[4]^ Seeking a second opinion may also cause patients to delay treatment.^[5]^ In Taiwan, a study by Huang et al^[6]^ showed lung cancer patients who had higher treatment refusal rates were associated with factors such as men, older adults, high prediagnosis Charlson comorbidity index (CCI) score, other catastrophic illnesses or injury, and advanced cancer stage. The National Health Insurance (NHI) Program covers 99.9% of the population and waives copayments for patients with cancer.^[7]^ The NHI Administration has also included 93% of Taiwan's health services organizations as NHI-contracted health care providers as of the end of 2014.^[7]^ Even given this ease of health care access and waiving of copayments for patients with cancer, a small proportion of Taiwanese patients who have been diagnosed with cervical cancer delay their treatment. The Taiwan government aims to decrease mortality from cervical cancer. In order to reduce cervical cancer mortality, we need to understand why some cervical cancer patients delayed or did not receive their cancer treatments. This study aimed to examine factors related to treatment delay in these cervical cancer patients and the effect on their survival.

## 2 Materials and methods

### 2.1 Data sources

Taiwan's NHI is a compulsory single-payer health insurance program that provides comprehensive coverage for medical care. Patient data were obtained from the Ministry of Health and Welfare's National Health Insurance Research Database and cause of death database, and the Health Promotion Administration's Cancer Registry files from 2005 through 2010. Diagnoses were coded according to the International Classification of Diseases for Oncology (ICD-O) from the Taiwan Cancer Registry. Since this study used the National Health Insurance Research Database and Cancer Registry Files, which were published by Taiwan government, the patient identification information has been deleted prior to analysis, and personal privacy was protected in this study. The study has been approved by the Institutional Review Board of China Medical University Hospital (IRB No.: CMUH102-REC3-076).

### 2.2 Patient selection and definition

A retrospective, longitudinal cohort study was carried out with patients who had a new diagnosis of cervical cancer between 2005 and 2010 and delayed treatment, followed up to the end of 2012 for survival analysis. There were 9081 patients with newly diagnosed cervical cancer which excluded stage 0 or stage missing, diagnosed after death, or other missing value in Taiwan from 2005 to 2010. For women with abnormal Pap test results, cervical cancer is diagnosed by either cervical biopsy of grossly visible lesions or colposcopically directed biopsy.^[8]^ Patient diagnoses were coded according to ICD-O, and cervical cancer cases were distinguished by codes corresponding to the relevant primary disease site C530 through C539. Treatment delay was defined as not undergoing any conventional cancer treatment (including surgery, radical surgery, radiation therapy, and chemoradiation)^[8]^ within 4 months of a confirmed diagnosis of cervical cancer. Data from the cancer treatment registry of the Health Promotion Administration indicate that the stage or condition of cancer changes in the absence of prompt treatment, and that 4 months postdiagnosis is the time by which the 93% of cancer patients who do undergo treatment have initiated treatment.^[9]^ For these reasons, patients who did not undergo treatment within 4 months of diagnosis^[9,10]^ were selected as study subjects for our analysis of treatment delay.

### 2.3 Measurements

Two outcomes were examined in this study: whether the cervical cancer patient delayed treatment past 4 months and whether the patient survived. The independent variables analyzed included demographic characteristics such as age at diagnosis, residence urbanization level^[11]^ (overall 7 levels; level 1 was the most urbanized and level 7 was the least urbanized), and socioeconomic status (monthly salary and insurance status), health status (comorbidity as CCI and presence of catastrophic illness or injury other than cancer), cancer stage (stages I to IV), and characteristics of the diagnosing hospital (hospital level and hospital ownership type). All cancer patients were identified in the NHI catastrophic illness or injury registry file, which contains 30 categories of patients with any severe illness or injury, such as cancer, chronic renal failure, systemic lupus erythematosus, type I diabetes, and so on, defined by the NHI.^[12]^ In National Cancer Registry, the clinical staging system (stages I to IV) in cervical cancer developed by the International Federation of Gynecology and Obstetrics (FIGO) which is the representative and associated with whole histologic types of cervical cancer.^[8]^ Deyo's adaptation of the CCI^[13]^ was used to assess the severity of comorbid conditions in cervical cancer patients, and the computed scores were classified into four categories: ≤3, 4–6, and ≥7. Taiwan's 359 townships and city districts were previously classified by Liu et al into 7 levels by urbanization, level 1 (most urbanized) through level 7 (least urbanized), representing highly urbanized cities, moderately urbanized cities, developing cities, average towns, aging towns, agricultural towns, and remote villages, respectively.^[14]^ The definition of level of urbanization has been widely used in relevant studies.^[6,9,15,16]^

### 2.4 Statistical analysis

Descriptive statistics were used to analyze, for each year from 2005 to 2010, the demographic characteristics and proportions of cervical cancer patients and of the subset who delayed treatment. Whether treatment delay was associated with patients’ demographic characteristics, socioeconomic status, health status, cancer stage, or diagnosing hospital characteristics was determined by the χ^2^ test and mean age at diagnosis was determined by *t*-test. To adjust for the cluster effects of patients having the same diagnosing hospital, logistic regression was performed using the generalized estimating equation model to analyze the association of factors with treatment delay in cervical cancer patients. In order to control all variables, we put all relative variables in the logistic model and the Cox proportional hazard model to control other variables completely.

To examine the effect of treatment delay on survival in cervical cancer patients, the log-rank test was used to first determine whether patient survival, followed up through 2012, correlated with demographic characteristics, socioeconomic status, health status, cancer stage, histologic type, or diagnosing hospital characteristics. Then the Cox proportional hazard model was used to evaluate the effect of treatment delay on patient survival after adjusting for all other related variables. Finally, factors affecting the survival of treatment-delayed patients were analyzed. All statistical analyses were performed with SAS software, version 9.3 (SAS Institute, Cary, NC).

## 3 Results

Between 2005 and 2010, a total of 9081 patients received a new diagnosis of cervical cancer in Taiwan, of whom 347 patients (3.82%) delayed treatment. Treatment delay declined annually during this period (Table 1), with the proportion of cervical cancer patients who delayed treatment decreasing from 6.46% in 2005 to 2.48% in 2010.

**Table 1:**
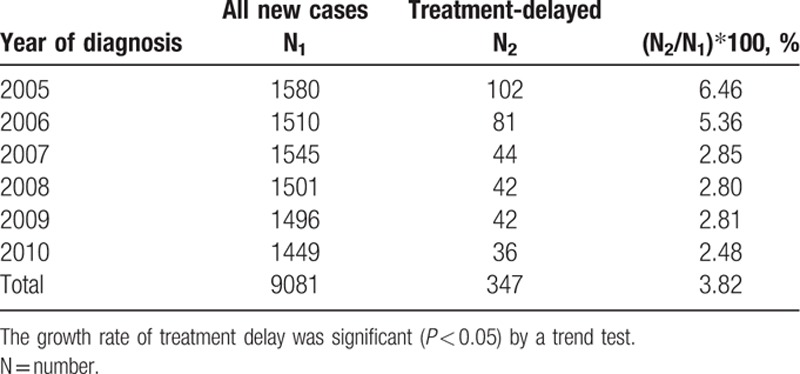
Rate of treatment delay for cervical cancer between 2005 and 2010.

### 3.1 Characteristics and treatment status of cervical cancer patients: factors associated with treatment delay

The characteristics of cervical cancer patients who delayed treatment were examined in terms of demographic characteristics, socioeconomic status, health status, cancer stage, and the diagnosing hospital characteristics (Table 2). With respect to patient mean age, treatment-delayed cervical cancer patients were on average 8.69 years older than treated patients (65.38 ± 17.27 years vs 56.69 ± 14.14 years; *P* < 0.05). Patients aged ≥75 years delayed treatment at the highest rate of all age categories, at 9.91% (*P* < 0.05). Areas classified as urbanization level 6, or agricultural towns, had the lowest treatment delay rate (3.48%) of all residence area categories. While controlling for the other variables in the model, the delay rates (3.49%) in urbanization level 2, or moderately urbanized cities, of residence areas was statistically significant difference (*P* < 0.05).

**Table 2:**
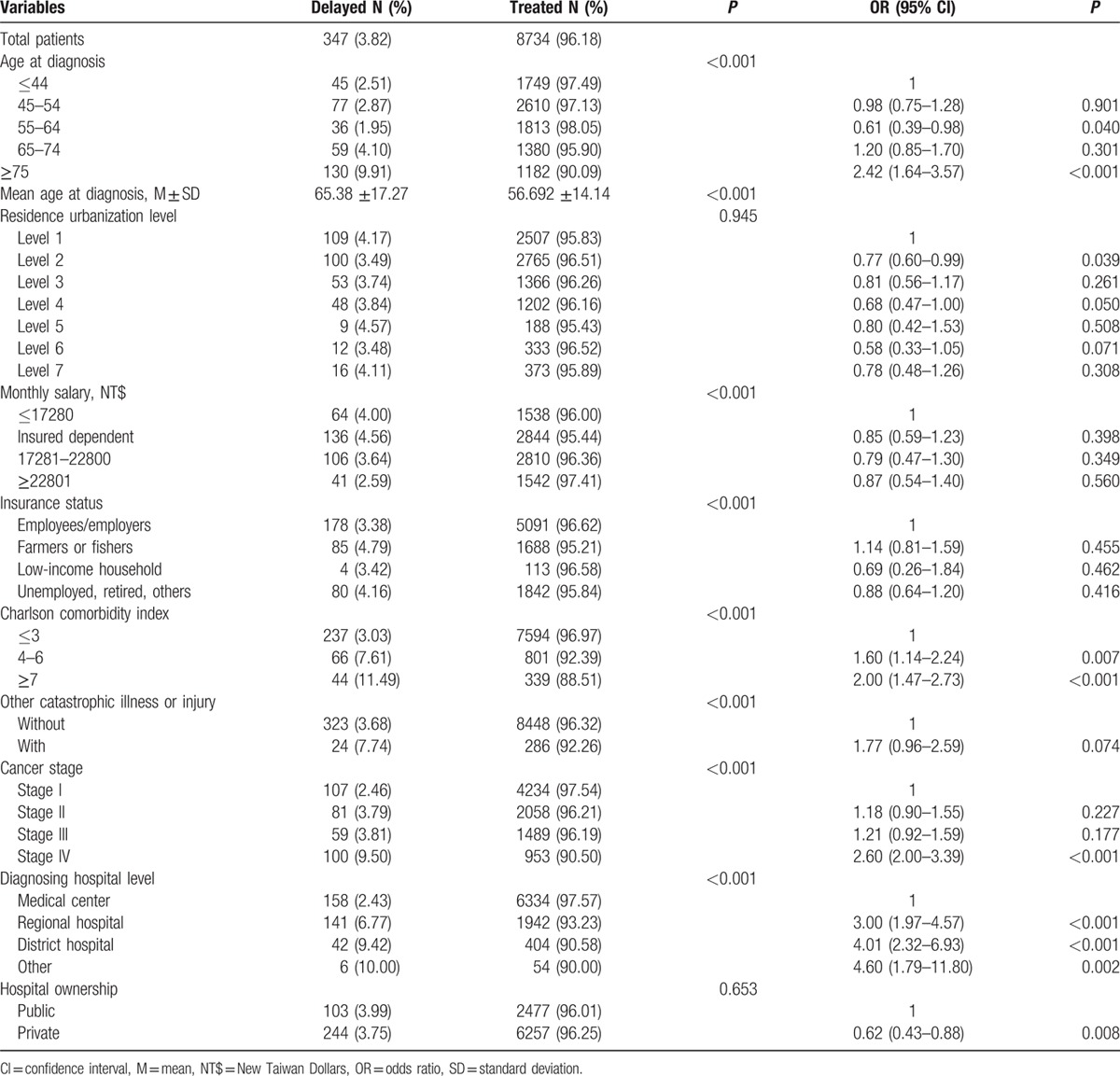
Characteristics and treatment status of cervical cancer patients: association of factors with treatment delay (generalized estimating equation model).

The rate of treatment delay in cervical cancer patients also varied with socioeconomic factors. With respect to income, the treatment delay rate would increase with decreasing monthly salary, but the difference among the participants with different monthly salary was not statistically significant (*P* > 0.05) after controlling for other variables. Among the insurance status categories, farmers or fishers had the leading rate of treatment delay of 4.79%, but the difference was not statistically significant (*P* > 0.05) after controlling for other variables.

In relation to patient health status, cervical cancer patients with higher CCI had higher treatment delay rate (*P* < 0.05). Patients with preexisting catastrophic illnesses or injuries also had a higher rate of treatment delay after cervical cancer diagnosis (7.74%), but the difference was not statistically significant (*P* > 0.05) after controlling for the variables. Of the cancer stages, stage IV corresponded to the highest treatment delay rate (9.50%, *P* < 0.05), and stage I, the lowest (2.46%, *P* < 0.05). With respect to the level of the diagnosing hospital, medical centers made more cervical cancer diagnoses and had the lowest rate of treatment delay (2.43%) among the diagnosed patients than other levels of hospitals (*P* < 0.05). In terms of the hospital ownership, the treatment delay rate at private hospitals was lower than the treatment delay rate at public hospitals (*P* < 0.05; Table 2).

### 3.2 Contributions of factors to treatment delay in cervical cancer patients

The risk factors for treatment delay in cervical cancer patients were analyzed by logistic regression. In order to take into consideration the cluster effects of patients having the same diagnosing hospital, a generalized estimating equation approach was used in the analysis. As shown in Table 2, patients of advanced age (≥75 years) had 2.42 times the odds of delaying treatment as that of patients ≤44 years of age (95% confidence interval [CI]: 1.64–3.57; *P* < 0.05). Also, patients with CCI scores of 4 to 6, ≥7 (OR = 1.6, 2; *P* < 0.05) had significantly higher odds of treatment delay than patients with CCI scores ≤3. With respect to cancer stage, the likelihood of treatment delay increased with increasingly advanced cancer stage. The odds for stage IV patients were 2.6 times (95% CI: 2.00–3.39; *P* < 0.05) that for the stage I. The patient's monthly salary, insurance status, residence urbanization level, and other catastrophic illnesses or injuries were not significantly associated with treatment delay (*P* > 0.05).

### 3.3 Effects of treatment delay and associated factors on survival in cervical cancer patients

The analysis on the effect of treatment delay on the survival of cervical cancer patients is presented in Table 3. After adjusting for other variables, treatment delay of 4 months or longer in cervical cancer patients was associated with a 2.31 times increased risk of death relative to timely treatment (95% CI: 2.01–2.65; *P* < 0.05). The effects of other factors on survival in treatment-delayed cervical cancer patients were also analyzed, one of the factors being age at diagnosis. Relative to the reference age group, namely patients aged ≤44 years, patients aged ≥75 years were 1.63 times as likely to die (95% CI: 1.42–1.87; *P* < 0.05). Whereas patients aged 55 to 64 years (adjusted hazard ratio [adj. HR] = 0.81, 95% CI: 0.71–0.94; *P* < 0.05) had a lower mortality risk than the reference group. Low-income household was at 1.47 times increased mortality risk relative to the employees/employers group (*P* < 0.05). The presence of other catastrophic illness or injury significantly affected risk of death in treatment-delayed cervical cancer patients (*P* < 0.05). Comorbidity in treatment-delayed cervical cancer patients was positively correlated with mortality risk. Relative to the reference group, CCI scores of ≤3, the adjusted hazard ratio for death increased from 5.86 for CCI scores of 4 to 6 to 10.67 for scores ≥7 (*P* < 0.05). Mortality risk also rose with increasingly advanced cancer stage. Relative to the reference group, cancer stage I, the adjusted hazard ratios for death for stages II, III, and IV respectively increased from 1.93, 2.81 to 5.19 (*P* < 0.05). The effects of histologic type on survival in treatment-delayed cervical cancer patients were analyzed. Adenocarcinoma in treatment-delayed cervical cancer patients was associated with a 1.24 times increased risk of death relative to squamous cell carcinoma (95% CI: 1.10–1.39; *P* < 0.05). The residence urbanization level, monthly salary, and hospital ownership did not significantly affect mortality risk in treatment-delayed cervical cancer patients (*P* > 0.05).

**Table 3:**
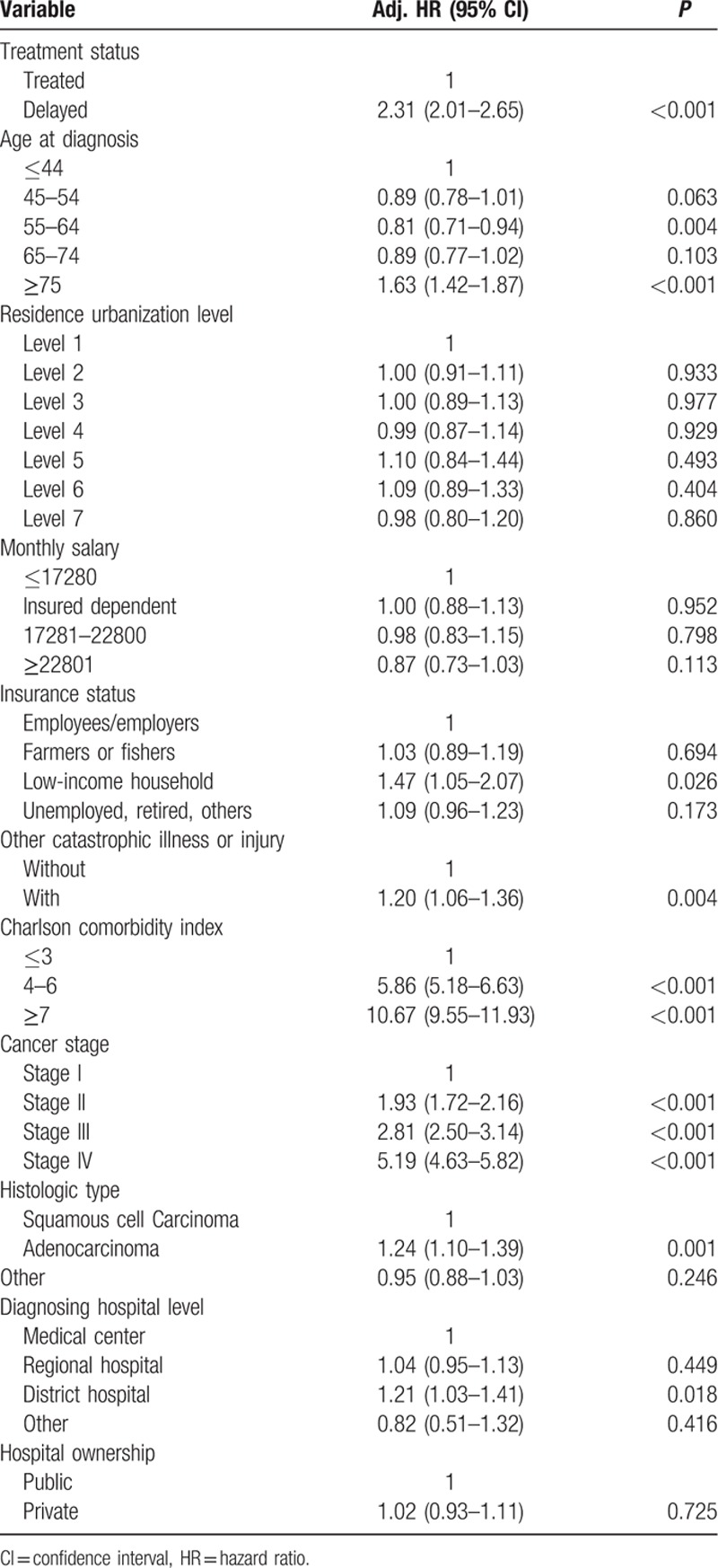
Cox proportional hazard model: correlation of factors with survival.

### 3.4 Survival rates for cervical cancer

**Figure 1:**
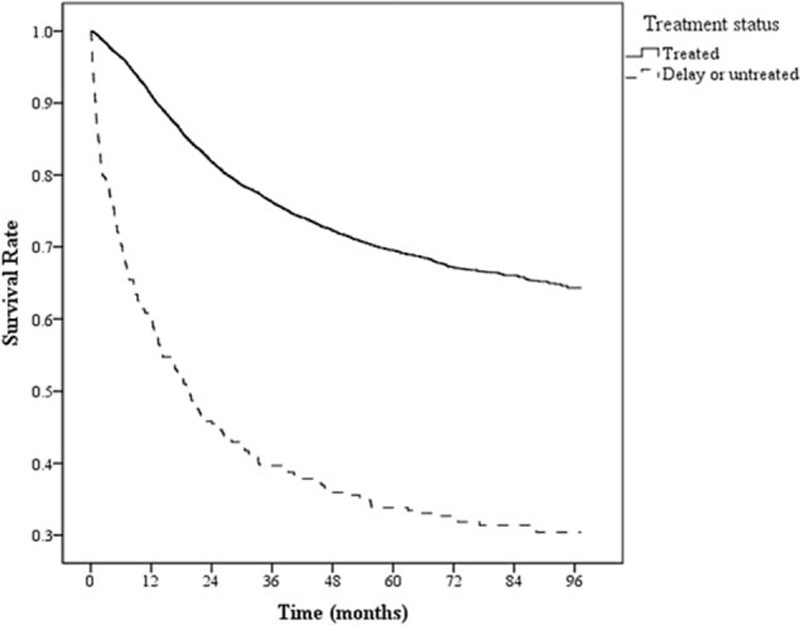
Survival curves of treated patients and delayed treatment patients for cervical cancer.

**Figure 2:**
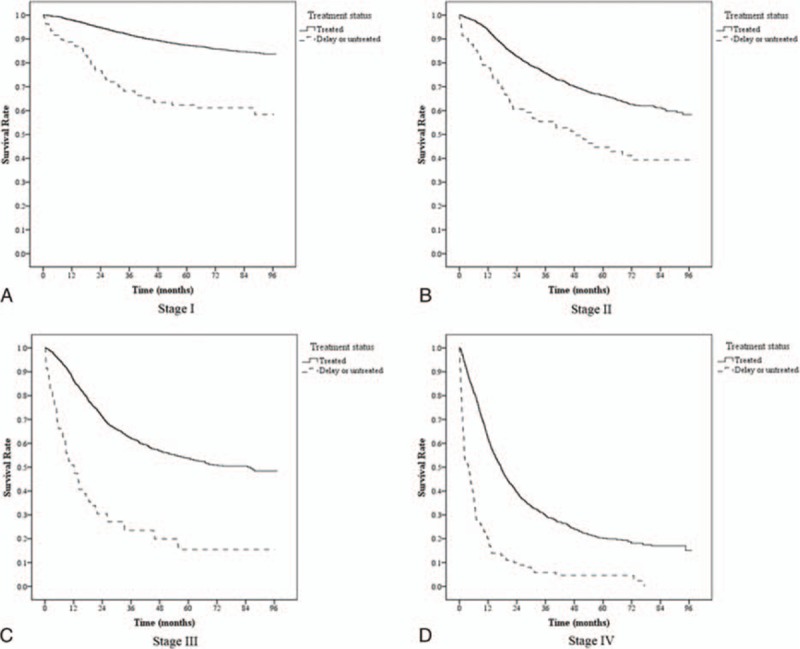
Survival curves of treated patients and delayed treatment patients for cervical cancer at different stages.

The treated cervical cancer patients had 2.46 times median follow-up time relative to the treatment-delayed cervical cancer patients (19.4 months; Table 4). Overall survival was compared for patients with a confirmed cervical cancer diagnosis who either received treatment within 4 months of diagnosis or delayed treatment. As shown by the Kaplan–Meier curves (Fig. 1), the gap in the 2 groups’ survival rates was significantly different (*P* < 0.05) and widened with time. The 1-year survival of treatment-delayed cervical cancer patients was 59.65%, significantly lower than that of treated patients, 90.90%. Survival decreased with increasing time to 37.92% for treatment-delayed patients and 70.75% for treated patients at 5 years (Table 5). We exhibited the survival curves of treated patients and delayed treatment patients for cervical cancer at different stages in Fig. 2.

**Table 4:**
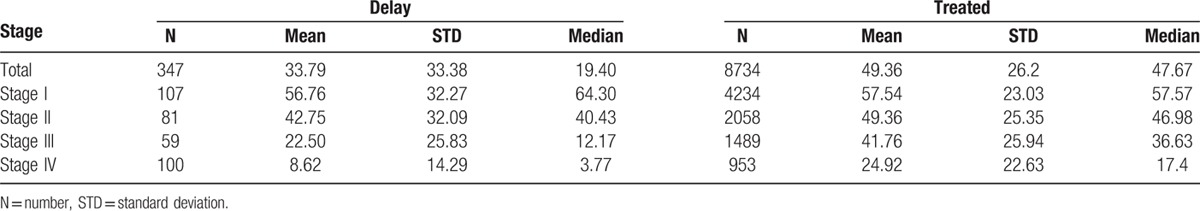
Average follow-up time (months) for the cervical cancer patients.

**Table 5:**
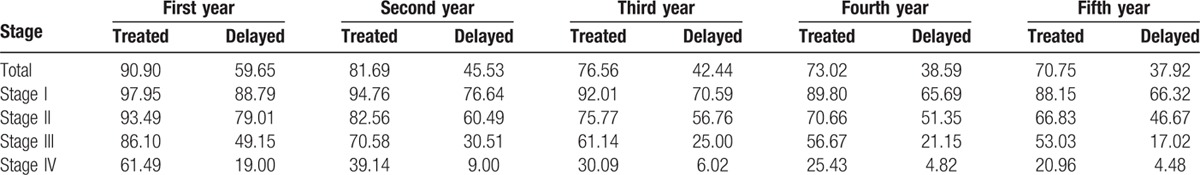
Survival (%) of treated patients and delayed treatment patients for cervical cancer.

## 4 Discussion

In our present analysis, the rate of treatment delay in Taiwanese patients with a new confirmed diagnosis of cervical cancer decreased from 6.46% to 2.48% between 2005 and 2010. A possible reason for this sharp decline in treatment delay is the implementation of the Five-Year National Program on Cancer Prevention and Control by Taiwan's Health Promotion Administration from 2005 to 2009.^[17]^ A key part of this plan was the 2005–2007 “cancer screening for early detection and early treatment” campaign, which aimed at promoting the Taiwanese population's awareness of early cancer detection and treatment.

Our analysis revealed that cervical cancer patients of more advanced age (≥65 years) at diagnosis were more likely to delay treatment (Table 2). Comparable observations have been made in other published studies that the rate of treatment refusal increased with increasing age of the cancer patient.^[18–22]^ In the study by Huchcroft and Snodgrass^[22]^ on Canadian patients who refused cancer treatment between 1975 and 1988, the authors concluded that the treatment refusal rate was higher in patients residing in remote areas because of these areas’ lower health care accessibility and less advanced treatment technologies relative to urban areas. In our study, after controlling for other variables in our regression model, we found the urbanization level of the residence area, except in urbanization level 2, or moderately urbanized cities, not to be a factor in whether the patient underwent treatment (*P* > 0.05; Table 2). This finding differs from those of past studies conducted in other countries, and may be a result of Taiwan's NHI system and medical outreach programs for remote areas.

With respect to economic factors, Lin et al^[23]^ in 2011 found that medication cost was one of the barriers to adherence to physician-prescribed therapy among breast cancer patients. In the United States, young women with public or no insurance and with low socioeconomic status tended to have longer treatment delay times after breast cancer diagnosis.^[24]^ Our analysis (Table 2) showed a lower rate of treatment delay in cervical cancer patients with higher monthly salary, but the association between salary and treatment delay was not statistically significant after controlling for related variables (*P* > 0.05). The difference between our finding and those of studies conducted in other countries can be explained by that under Taiwan's NHI program, the government subsidizes the insurance premiums of the economically disadvantaged, thus lowering the barriers to medical care for low-income individuals and minimizing the effect of income on treatment seeking or delay.

Previous studies have identified a number of factors that influenced treatment refusal or discontinuation by cancer patients. These factors include having multiple cancers,^[22]^ an advanced cancer stage, or worsening disease;^[21]^ patients’ state of health, access to pertinent information, attitude toward their disease, interaction with and encouragement from health care staff, and concern about adverse effects of treatment.^[25]^ In a 2012 study on breast cancer patients who refused conventional treatment and opted for alternative therapy, Citrin et al^[11]^ showed that negative perceptions of physician demeanor (uncaring, insensitive, and unnecessarily harsh), fear of side effects, belief in the efficacy of alternative therapies (including consumption of raw fruits and vegetables and supplements) were factors in the patients’ treatment refusal. The reasons of nontreatment or interrupted treatment within 4 months among breast, colon, oral, and cervical cancer patients were studied in Tsai et al questionnaire study. The main points for treatment delay of cervical cancer patients are the fear of surgery, economic burden of household, worried about poor life quality after therapy, and no companions for treatment.^[9]^ According to the results of our regression analysis (Table 2), the likelihood of treatment delay in cervical cancer patients significantly increased with increasingly advanced cancer stage and was significantly higher for diagnoses made at nonmedical center hospitals. Huchcroft and Snodgrass^[22]^ also showed that besides patients with advanced cancer, some of the treatment refusers were cancer patients who rejected not only cancer treatment but also further testing for cancer staging, and were consequently designated as having unstaged disease. In our present study, there were similar cases of cervical cancer with unspecified stage, which may indicate that a proportion of the patients did not wish to undergo further testing for cancer stage determination.

Treatments of cervical cancer depend on stage of disease. Stage I to IIA (early stage) can be treated with either radical surgery or radiation therapy. Stages IIB to IV (advanced stage) should be treated with chemoradiation and radiation therapy.^[8]^ The treatment decision lies with the gynecologist and should be based on a review of the specimen with the pathologist. Studies have shown that longer waiting times from diagnosis to treatment and from the initial visit to surgical intervention in cervical cancer patient were not associated with worse survival and outcome of cervical cancer patients.^[26,27]^ More specifically, cancer survival has been shown to be affected by different factors. Coker et al^[28]^ analyzed 1251 cervical cancer patients identified between 1992 and 1999, and found poorer survival rates in patients with more advanced age, more advanced cancer stage, and greater comorbidity. Choan et al^[29]^ found that longer waiting times of cervical cancer patients treated with radical radiotherapy were associated with reduced survival outcomes. In Sant et al's^[30]^ analysis of 5-year relative survival in breast cancer patients, the relative excess risk for tumor stage IV was 3.38 times that for stage I. Ma et al's^[31]^ study on non-small-cell lung cancer patients also identified cancer stage to be a main factor affecting the patients’ survival. We also found that, relative to timely treatment, treatment delay lowered the 1- to 5-year survival rates and shortened mean survival time in cervical cancer patients (Fig. 1), which is analogous to Liu et al's^[32]^ 2014 finding in untreated colorectal cancer patients.

## 5 Conclusions

Under Taiwan's universal health insurance system, compulsory health insurance enrollment, along with medical outreach efforts in remote mountainous areas, has lowered barriers and enhanced access to medical care for the Taiwanese population. The results of our present analysis show that among Taiwanese patients with a new confirmed diagnosis of cervical cancer between 2005 and 2010, the rate of treatment delay of 4 months or longer exhibited a yearly decline during that period. This study can help identify cervical cancer patients at risk of delay. Higher rates of treatment delay were found in cervical cancer patients characterized by more advanced age, more severe comorbidity, more advanced cancer stage, having a nonmedical center diagnosing hospital, or diagnosing at public hospital ownership. Not undergoing treatment within 4 months was associated with a 2.31-fold increased risk of death relative to timely treatment. Cervical cancer patients with more advanced age, low-income household, other catastrophic illness or injury, higher comorbidity, more advanced cancer stage, adenocarcinoma, or diagnosing at district hospital level had a higher risk of death. Because the present analysis was performed with secondary databases as the retrospective data source, the factors involved in treatment delay in Taiwanese cervical cancer patients could not be clarified fully if this was due to doctor's delay, intentional or by mistake, patients’ decision, failure to seek treatment or inability to start treatment due to poor surgical candidacy, or inability to tolerate chemoradiation. In addition, the database lacked information on patient's marital status, education level, lifestyle, health behaviors, medical knowledge, physical and psychological status, and family care and support. A more in-depth understanding of this problem awaits further research by means of questionnaires or interviews.

## Acknowledgments

We are grateful for use of the National Health Insurance Research Database and the Cancer Register Files provided by Statistic Center of Department of Health and Welfare, Taiwan.

## References

[1] UK CR. Cervical cancer mortality statistics. 2014 http://www.cancerresearchuk.org/health-professional/cancer-statistics/statistics-by-cancer-type/cervical-cancer Accessed Nov 1, 2015.

[2] Global cancer burden rises to 14.1 million new cases in 2012: marked increase in breast cancers must be addressed (press release). International Agency for Research on Cancer 2013.

[3] Health Promotion Administration. 2014 Health Promotion Administration Annual Report. Taiwan: Ministry of Health and Welfare; 2014.

[4] Hunter MI Tewari K Monk BJ Cervical neoplasia in pregnancy. Part 2: current treatment of invasive disease. *Am J Obstet Gynecol* 2008; 199:10–18. 18585521 10.1016/j.ajog.2007.12.011

[5] Support MC. Getting a second opinion. 2012 http://www.macmillan.org.uk/Cancerinformation/Cancertreatment/Gettingtreatment/Gettingasecondopinion.aspx Accessed Nov 15, 2015.

[6] Huang HL Kung PT Chiu CF Factors associated with lung cancer patients refusing treatment and their survival: a national cohort study under a universal health insurance in Taiwan. *PLoS One* 2014; 9:e101731. 24999633 10.1371/journal.pone.0101731 PMC4084901

[7] National Health Insurance Administration. National Health Insurance in Taiwan 2014-2015 Annual Report. Taiwan: Ministry of Health and Welfare; 2015.

[8] Berek JS Berek & Novak's Gynecology. 15th ed. Sun Valley, NV, U.S.A.: Lippincott Williams & Wilkins, a Wolters Kluwer Business; 2012.

[9] Tsai WC Exploring the Reasons of Non-treatment for Cancer Patients in Taiwan. Taiwan: Ministry of Health and Welfare; 2013.

[10] Health Promotion Administration. Taiwan Cancer Registry Long Form Excerpt Manual. Taiwan: Ministry of Health and Welfare; 2013.

[11] Citrin DL Bloom DL Grutsch JF Beliefs and perceptions of women with newly diagnosed breast cancer who refused conventional treatment in favor of alternative therapies. *Oncologist* 2012; 17:607–612. 22531358 10.1634/theoncologist.2011-0468 PMC3360900

[12] Categories of Catastrophic Illness or Injury. Taiwan: Ministry of Health and Welfare; 2011.

[13] Deyo RA Cherkin DC Ciol MA Adapting a clinical comorbidity index for use with ICD-9-CM administrative databases. *J Clin Epidemiol* 1992; 45:613–619. 1607900 10.1016/0895-4356(92)90133-8

[14] Liu CY Hung YT Chuang YL Incorporating development stratification of Taiwan townships into sampling design of large scale health interview survey. *J Health Manag* 2006; 4:1–22.

[15] Hsu YH Tsai WC Kung PT Health examination utilization in the visually disabled population in Taiwan: a nationwide population-based study. *BMC Health Serv Res* 2013; 13:509. 24313981 10.1186/1472-6963-13-509 PMC3880214

[16] Chen SJ Kung PT Huang KH Characteristics of the delayed or refusal therapy in breast cancer patients: a longitudinal population-based study in Taiwan. *PLoS One* 2015; 10:e0131305. 26114875 10.1371/journal.pone.0131305 PMC4482743

[17] Health Promotion Administration. Cancer prevention and control. 2015 http://www.hpa.gov.tw/English/ClassShow.aspx?No=201401280006 Accessed Jan 1, 2016.

[18] Jassem J Ozmen V Bacanu F Delays in diagnosis and treatment of breast cancer: a multinational analysis. *Eur J Public Health* 2013. 10.1093/eurpub/ckt131 24029456

[19] Germann N Haie-Meder C Morice P Management and clinical outcomes of pregnant patients with invasive cervical cancer. *Ann Oncol* 2005; 16:397–402. 15668263 10.1093/annonc/mdi084

[20] Ward MM Ullrich F Matthews K Who does not receive treatment for cancer. *J Oncol Pract* 2013; 9:20–26. 23633967 10.1200/JOP.2012.000829 PMC3545658

[21] Konigsberg RD The refuseniks. *Time* 2011; 177:72–74.77. 21682135

[22] Huchcroft SA Snodgrass T Cancer patients who refuse treatment. *Cancer Causes Control* 1993; 4:179–185. 8318634 10.1007/BF00051311

[23] Lin JH Zhang SM Manson JE Predicting adherence to tamoxifen for breast cancer adjuvant therapy and prevention. *Cancer Prev Res (Phila)* 2011; 4:1360–1365. 21893499 10.1158/1940-6207.CAPR-11-0380

[24] Smith EC Ziogas A Anton-Culver H Delay in surgical treatment and survival after breast cancer diagnosis in young women by race/ethnicity. *JAMA surgery* 2013; 148:516–523. 23615681 10.1001/jamasurg.2013.1680

[25] Sainio C Lauri S Eriksson E Cancer patients’ views and experiences of participation in care and decision making. *Nurs Ethics* 2001; 8:97–113. 16010885 10.1177/096973300100800203

[26] Perri T Issakov G Ben-Baruch G Effect of treatment delay on survival in patients with cervical cancer: a historical cohort study. *Int J Gynecol Cancer* 2014; 24:1326–1332. 25054445 10.1097/IGC.0000000000000211

[27] Umezu T Shibata K Kajiyama H Prognostic factors in stage IA-IIA cervical cancer patients treated surgically: does the waiting time to the operation affect survival? *Arch Gynecol Obstet* 2012; 285:493–497. 21735188 10.1007/s00404-011-1966-y

[28] Coker AL Du XL Fang S Socioeconomic status and cervical cancer survival among older women: findings from the SEER-Medicare linked data cohorts. *Gynecol Oncol* 2006; 102:278–284. 16434087 10.1016/j.ygyno.2005.12.016

[29] EC Dahrouge S Samant R Mirzaei A Radical radiotherapy for cervix cancer: the effect of waiting time on outcome. *Int J Radiat Oncol Biol Phys* 2005; 61:1071–1077. 15752886 10.1016/j.ijrobp.2004.09.030

[30] Sant M Allemani C Capocaccia R Stage at diagnosis is a key explanation of differences in breast cancer survival across Europe. *Int J Cancer* 2003; 106:416–422. 12845683 10.1002/ijc.11226

[31] Ma H Shu Y Pan S Polymorphisms of key chemokine genes and survival of non-small cell lung cancer in Chinese. *Lung Cancer* 2011; 74:164–169. 21514686 10.1016/j.lungcan.2011.03.005

[32] Liu CY Chen WT Kung PT Characteristics, survival, and related factors of newly diagnosed colorectal cancer patients refusing cancer treatments under a universal health insurance program. *BMC Cancer* 2014; 14:446. 24938667 10.1186/1471-2407-14-446 PMC4072493

